# Psychological Impact of COVID-19 Outbreak on Families of Children with Autism Spectrum Disorder and Typically Developing Peers: An Online Survey

**DOI:** 10.3390/brainsci11060808

**Published:** 2021-06-18

**Authors:** Annalisa Levante, Serena Petrocchi, Federica Bianco, Ilaria Castelli, Costanza Colombi, Roberto Keller, Antonio Narzisi, Gabriele Masi, Flavia Lecciso

**Affiliations:** 1 Department of History, Society, and Human Studies, University of Salento, 73100 Lecce, Italy; flavia.lecciso@unisalento.it; 2 Laboratory of Applied Psychology, Department of History, Society, and Human Studies, University of Salento, 73100 Lecce, Italy; serena.petrocchi@usi.ch; 3 Faculty of Biomedical Sciences, Università della Svizzera Italiana, Via Buffi 13, 6900 Lugano, Switzerland; 4 Department of Human and Social Sciences, University of Bergamo, 23129 Bergamo, Italy; Federica.bianco@unibg.it (F.B.); ilaria.castelli@unibg.it (I.C.); 5 IRCCS Stella Maris Foundation, 56018 Pisa, Italy; costanza.colombi@fsm.unipi.it (C.C.); antonio.narzisi@fsm.unipi.it (A.N.); gabriele.masi@fsm.unipi.it (G.M.); 6 Department of Psychiatry, University of Michigan, Ann Arbor, MI 48109, USA; 7 Adult Autism Center, Mental Health Department, Local Health Unit ASL Città di Torino, 10138 Turin, Italy; roberto.keller@aslcittaditorino.it

**Keywords:** COVID-19, autism spectrum disorder, survey, children, lockdown, parental distress, emotion responses, adaptive behavior, typically developing, mediation

## Abstract

Background: When COVID-19 was declared as a pandemic, many countries imposed severe lockdowns that changed families’ routines and negatively impacted on parents’ and children’s mental health. Several studies on families with children with autism spectrum disorder (ASD) revealed that lockdown increased the difficulties faced by individuals with ASD, as well as parental distress. No studies have analyzed the interplay between parental distress, children’s emotional responses, and adaptive behaviors in children with ASD considering the period of the mandatory lockdown. Furthermore, we compared families with children on the spectrum and families with typically developing (TD) children in terms of their distress, children’s emotional responses, and behavioral adaptation. Methods: In this study, 120 parents of children aged 5–10 years (53 with ASD) participated. Results: In the four tested models, children’s positive and negative emotional responses mediated the impact of parental distress on children’s playing activities. In the ASD group, parents reported that their children expressed more positive emotions, but fewer playing activities, than TD children. Families with children on the spectrum reported greater behavioral problems during the lockdown and more parental distress. Conclusions: Our findings inform the interventions designed for parents to reduce distress and to develop coping strategies to better manage the caregiver–child relationship.

## 1. Background

A huge amount of literature has examined general distress in families with children with autism spectrum disorder (ASD) [1]. Evidence highlights that in these families, parental distress is higher compared to parents of children born without any developmental disorder [2,3], or even those with other developmental disorders [4,5,6,7]. There are several known factors that increase parental distress and the probability of developing mental health problems [8]. One factor concerns the burden experienced by the caregivers, which consists of handling children’s behavioral problems in terms of impulsive behaviors, rigidity, sameness, and sensory concerns [9,10,11,12]. Emotional regulation is another critical area of functioning for children with ASD, with negative impact on the caregiving system [13,14,15], together with the management of children’s food- and sleep-related routines [16]. Another factor that may boost parental distress is the severity of ASD—high- vs. low-functioning—and the children’s relative cognitive and social impairment [17,18]. Additionally, parents’ awareness that their children’s autistic traits may be persistent and lifelong can further increase their distress [19]. Poor availability of social support received from qualified health and educational professionals can also be relevant for parental distress [20,21]—particularly after the first diagnosis, during the reaction process to the diagnosis [22,23]. It has been found that stress and anxiety are higher when social support is lacking [24], and this negatively affects parental coping abilities [25]. Finally, studies on the effects of children’s gender and age on parental distress provide mixed evidence. Several authors [26,27] suggest that having younger male children with ASD significantly and negatively influences the distress experienced by parents, whereas other works [26,28] find that children’s behavioral problems have an impact on parental stress regardless of gender and age. Therefore, the challenges of caring for a child with ASD place significant distress on parents [29] and, in turn, their distress might affect the quality of the relationship with their own child(ren) and their behaviors [30].

## 2. The Impact of COVID-19 on Mental Health

When COVID-19 was declared a pandemic, the World Health Organization [31] suggested that all countries implement several public health measures to contain the spread of the virus and to protect their citizens [32], e.g., social/physical distancing, hygiene procedures, and mask protection. As a result of the mandatory lockdowns, schools, and health/educational centers, as well as recreational/sport centers, were closed. The lockdown and the social isolation because of the COVID-19 virus led the general population to experience intense distress [33,34], as well as exacerbating the symptomatology of psychiatric disorders [35]. Parental anxiety and worries regarding their own work (e.g., layoffs, pay cuts, or working from home), financial instability, the fear of the contagiousness of the disease [36], the limited access to health care services, and online learning conditions had an impact on caregiving skills, impairing parents’ ability to deal with their children during the emergence of COVID-19 [37]. Thus, the lockdown significantly changed families’ routines, and negatively impacted on parents’ and children’s mental health [38,39]. 

Hence, research has devoted special attention to the vulnerability of children with special needs [40,41], such as subjects with ASD [42], because of the disruption to daily life routines and social isolation [43], as it is well known that individuals with ASD are highly sensitive to unpredictable changes [44]. 

The COVID-19 outbreak rapidly created an unstable situation, which may have intensified autistic symptomatology and behavioral problems through the increase of parental distress. One study [41] on children with special needs, including children with ASD, revealed a high prevalence of distress in parents, and an association of this with the availability of online support from health care professionals. In another study [45], parents reported that their children with ASD expressed generally good management during the lockdown, with high levels of autonomy and communication with them, but they also observed the development of new stereotyped behaviors. Although the authors did not evaluate the possible associations, they also found that the majority of the families received adequate support from their psychologists or special needs teachers. Several other studies investigated the psychological impact of COVID-19 on the mental health of parents and/or children with ASD. Two of these [46,47] involved the Saudi population, whereas the others recruited Italian [48] or American parents [49]. 

The study by Alhuzimi [46] aimed to examine the impact of COVID-19, in terms of the changes to daily routines, on 150 parents (children’s age range = 2–18 years) and found that having a younger child and a greater severity of symptoms were the two factors most closely related to high parental distress and low wellbeing. Furthermore, parental distress was negatively affected by the lack of social support received from special education teachers and therapists. The study by Althiabi ([47]; *n* = 211 parents; children’s age range = 3–17 years) suggested that high parental anxiety and poor caregiving attitudes, together with the lack of social support and the severity of the symptomatology, increased parental distress. Manning et al. [49] found high levels of distress both in parents and in individuals with ASD—parent reported—(*n* = 471 parents; children’s age range = up to and beyond 21 years) caused by social isolation, fear of contagiousness, finance problems, and children’s behavioral problems. 

Finally, Colizzi et al. [48] investigated whether the clinical characteristics of individuals with ASD (*n* = 527 parents; children’s mean (sd) of age = 13 (8.1) years) negatively impacted on their wellbeing during the lockdown. They found that pre-existing behavioral problems predicted a higher risk of intense and frequent symptoms. Furthermore, the authors revealed that parents claimed to have had difficulties in managing their children’s daily free and structured activities. 

All these studies reveal that lockdowns have had an effect in terms of increasing difficulties among individuals with ASD, and show significant associations between parental distress, social support received, and children’s age and level of functioning. To the best of our knowledge, no studies have drawn attention to the interplay between parental distress, children’s emotional responses, and adaptive behaviors in children with autism during periods of mandatory lockdown. According to the tripartite model of Morris et al. [50], parents may affect their children’s emotional adaptation through imitation, the general affective environment experienced at home, and the strategies they use to respond to their children’s emotions. Therefore, when the contextual conditions become stressful—such as during lockdown—parents’ emotions, especially the negative ones, reverberate in children via the contagion mechanism [51]. The way in which parents deal with the stressful situation is then essential for their children to regulate themselves and to cope with the stress. Moreover, parental distress interferes with the caregiver system and, as a consequence, children can manifest emotional and behavioral problems [52]. Based on these considerations, in the present study, we aimed to explore the impact of parental distress on children’s adaptive behavior, as well as the mediation role of children’s emotional responses, in a sample of families with children with ASD aged 5–10 years, compared to a sample of typically developing children. In particular, the following hypothesis has been formulated:

**Hypothesis** **1** **(H1).** *We expected that parental distress during the COVID-19 lockdown would affect children’s adaptive behavior via the mediation role of the children’s positive (Model 1) and negative (Model 2) emotional responses, as perceived by parents, controlling for several covariates.*

**Figure 1:**
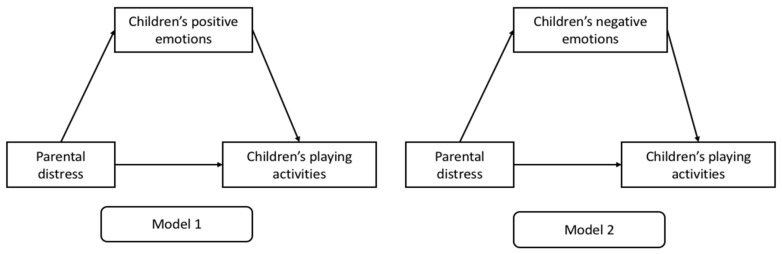
The hypothesized mediation models.

Figure 1 shows the mediation models hypothesized.

Furthermore, because lockdown led to the interruption of therapeutic and educational treatments, and to the disruption of children’s routines, we aimed at exploring children’s behavioral problems. Thus, our first research question was formulated: are there any differences between children’s behavioral problems (e.g., stereotypes, repetitive behaviors, aggressive behaviors, etc.) before and during the lockdown? (RQ1). 

Finally, to the best of our knowledge, no previous studies have compared the levels of parental distress and children’s outcomes during the lockdown in families with children with ASD and those with typically developing (TD) children. A second research question was thus: are there any differences in parental distress levels, in their perception of children’s positive and negative emotional responses, and in adaptive behavior when comparing families with children on the spectrum and those with typically developing children? (RQ2). Furthermore: are there any differences between the above hypothesized models? (RQ3).

## 3. Materials and Methods

### 3.1. Design and Procedure

Data were collected during the first mandatory lockdown due to COVID-19 in Italy in 2020. In both cases, an online parent-reported survey was implemented, wherein we asked parents to retrospectively think about the same lockdown period (April–May 2020) in which they were forced to stay at home. Qualtrics XM software was used to create the online survey, which was disseminated through the main social media platforms (i.e., WhatsApp, Facebook) and via snowball sampling technique. The two surveys were similar except for a part with specific questions for parents of children with an ASD.

Predefined inclusion criteria were: (1) being a parent of a child with an ASD diagnosis aged from 5 to 10 years or being a parent of a typically developing child aged from 5 to 10 years; (2) parental ability to read and understand the Italian language; (3) being an Italian citizen. The exclusion criteria were the presence of any pre-existing medical condition for the parents who completed the survey and for the children within the TD group. For debriefing, a list of suggestions to manage the caregiver-child relationship during the lockdown was given to both groups. The University Ethical Committee approved the research. Parents read an information sheet and electronically signed the consent form before their participation.

### 3.2. Participants

One hundred and twenty parents participated in the research. Fifty-three of them were parents of children with a self-declared ASD diagnosis—forty-four of whom were mothers and nine of whom were fathers. Their mean age was 41.8 years (SD = 5.4 years). Sixty-seven parents with typically developing children also participated; fifty-nine of them were mothers and eight were fathers. They had a mean age of 40.2 years (SD = 6.5 years). Table 1 reports the description of the sample’s characteristics and the results of the comparisons between the two groups.

### 3.3. Measures

*Parental Exposure to COVID-19*: In our survey we included five questions investigating parental exposure to the COVID-19 pandemic [34,53]. We asked parents whether they, their partner, their relatives, or their friends had tested positive for COVID-19 or shown similar symptoms. Finally, we included a question asking whether any of their relatives or friends had died because of COVID-19. Each question was worth 1 point if the participants/partner/relative/friend had tested positive for COVID-19 or had related symptoms, whereas they scored 0 points if they tested negative. Regarding the question asking whether someone had died because of COVID-19 infection, it scored 1 point if yes, and 0 if no. A cumulative score was obtained as the sum of the items (M = 0.45; sd = 0.82; range 0–5), with higher scores indicating greater exposure.

*Maternal distress*: Parental distress was assessed using the 21-item, self-reported Depression, Anxiety, and Stress Scale questionnaire (henceforth DASS-21; [54]). The DASS-21 evaluates distress as a composite construct that includes depressive, anxious, and stress symptoms. For example, depressive symptoms are evaluated by items such as “I felt discouraged and depressed” (item 13); anxiety is measured by items such as “I felt scared for no reason” (item 20); and, finally, stress levels are evaluated by items such as “I felt stressed” (item 11). The questionnaire allows measurement of the adults’ distress over the previous 7 days, which in the present study corresponded to the lockdown. Response options ranged from 0 (“never happened”) to 3 (“always happened”). The total distress score was calculated as the average of all items, with higher scores indicating higher distress levels (α = 0.92; rs ≥ 0.24). 

*Children’s emotional responses and adaptive behavior*: Seven ad hoc questions asked the parents to say how much their children showed positive (happy, quiet, and secure) and negative (sad, anxious, worried, and angry) emotions during the lockdown period [34]. One additional question, created ad hoc for this research, asked parents to say how much the children were involved in playing activities compared to usual during the lockdown. The response options ranged from 0 (“not at all”) to 4 (“very much”). For the children’s emotional responses, one score for positive and one for negative emotions were calculated as means of the related items, with higher scores indicating higher experience of positive (α = 0.88; rs ≥ 0.69) or negative (α = 0.88; rs ≥ 0.58) emotions. 

*Behavioral problems before and during the lockdown*: To explore behavioral problems in children with ASD before and during the lockdown, five ad hoc questions were formulated. Each question asked parents how the children’s stereotypes (“Did your child show stereotypes before/during the lockdown?”), their aggressive behaviors (“Was your child aggressive towards him/herself before/during the lockdown?”; “Was your child aggressive towards others before/during the lockdown?”), their repetitive behaviors (“Did your child show repetitive behaviors before/during the lockdown?”), and their ability to manage the transition from one activity to another (“Had your child difficulties in managing the transition from one activity to another before/during the lockdown?”) changed before and during the lockdown. Response options ranged from 0 (“not at all”) to 7 (“very much”). 

*Covariates*: In Models 1 and 2, several variables were used as covariates: children’s age, gender, and level of ASD functioning (high vs. low), as well as the number of professionals who supported the child (ranging from 0—no one supported the child during the lockdown—through 1—either the special education teacher or the therapist supported the child—to 2—both the special educational teacher and the therapist supported the child), and the frequency of the sport activities practiced by children during the lockdown (from 1 (“not at all”) to 4 (“more than usual”)). Furthermore, in Models 3 and 4 the children’s group (ASD vs. TD) was used as a covariate.

### 3.4. Statistical Analyses

The statistical analyses were performed using SPSS version 25. (IBM, Armonk, NY, USA). To evaluate the associations between variables, Spearman’s rho correlations were carried out. Mediations were performed using Process v3.0 (https://www.processmacro.org/index.html, accessed on 10 June 2021), applying Model 4 and 5000 bootstraps inference for model coefficients. In the mediation models, the predictor variable (x) was parental distress, the outcome (y) was children’s playing activities, and the mediators (M) were children’s emotional positive (Model 1 and Model 3) or negative (Model 2 and Model 4) responses, as reported by parents. Children’s age, gender, and ASD functioning level (high vs. low), as well as parental exposure to COVID-19, the social support received, the sport activities practiced by children, and the group (ASD vs. TD), were used as covariates. Paired sample *t*-tests were computed to compare children’s behavioral problems before and during the lockdown. Independent sample *t*-tests were used to compare parental distress and children’s emotional responses and adaptive behaviors in the ASD and TD groups.

## 4. Results

### 4.1. Correlations and Mediation in the ASD Sample 

Spearman’s correlations showed that parental distress significantly and negatively correlated with children’s positive emotions (*r* = −0.391; *p* = 0.004) and playing activities (*r* = −0.363; *p* = 0.007), as high distress levels indicate lower parental ability to perceive children’s positive emotions and their ability to play as usual during the lockdown. On the other hand, parental distress was significantly and positively correlated with their perception of their children’s negative emotions (*r* = 0.529; *p <* 0.001); in other words, distressed parents perceived their children as more sad, angry, worried, and anxious during the lockdown. The expected correlations emerged between children’s positive (*r* = 0.467; *p* < 0.001) and negative (*r* = −0.460; *p* = 0.001) emotions and children’s adaptive behavior, i.e., playing as usual.

**Figure 2:**
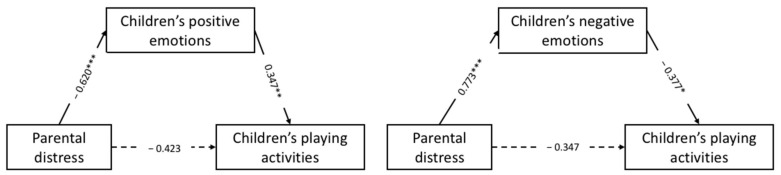
Models testing the mediation relationships. * *p* < 0.05; ** *p* < 0.01; *** *p* < 0.001.

The hypothesized models in Figure 2 were tested with children’s age, gender, and functioning (high vs. low)—as well as parental exposure to COVID-19, the social support received, and the sport activities practiced by children—as covariates. For both mediation models estimated, the indirect effects were significant. In other words, parental distress was associated with children’s tendency to involve themselves in their usual playing activities during the lockdown, through the mediation effects of the parental ability to detect children’s positive and negative emotional responses. 

Regarding Model 1 (F(5, 47) = 2.782; *p* = 0.028; R = 0.48; R^2^ = 0.23), parental distress was not significantly related to the children’s tendency to play as usual (β = −0.423; *p* > 0.05; BootLLCI = −0.877; BootULCI = 0.149). Distress was significantly and negatively related to children’s positive emotions (β = −0.610; *p* = 0.001; BootLLCI = −0.964; BootULCI = −0.234), indicating that increased parental distress levels were associated with lower perception by parents of their children’s positive emotions. Furthermore, children’s positive emotions were significantly and positively related to their adaptive behavior (β = 0.347; *p* = 0.046; BootLLCI = 0.079; BootULCI = 0.764), indicating that children’s increased positive emotions were associated with their greater tendency to play as usual. For Model 1, the total mediation effect of parental distress on children’s adaptive behavior mediated by children’s positive emotions was β = −0.638, *p* = 0.004 (BootLLCI = 1.068; BootULCI = −0.208). Finally, children’s functioning (β = −0.391; *p* = 0.048; BootLLCI = −0.788; BootULCI = −0.022) and their sport practice during the lockdown (β = 0.269; *p <* 0.001; BootLLCI = 0.148; BootULCI = 0.372) were significantly related to the children’s positive emotions. Specifically, children’s low functioning was associated with lower levels of positive emotions. Increased sport activities during the lockdown were associated with greater levels of positive emotions in children. The other covariates used in the model were not significantly correlated with the mediator or the outcome. 

Regarding Model 2 (F(5, 47) = 2.782; *p* = 0.028; R = 0.48; R^2^ = 0.23), parental distress was not significantly related to the children’s outcomes (β = −0.347; *p* > 0.05; BootLLCI = −0.803; BootULCI = 0.290). Parental distress was significantly and positively related to children’s negative emotions (β = 0.773; *p <* 0.001; BootLLCI = 0.431; BootULCI = 1.126), indicating that increased parental distress levels were associated with greater perception by parents of their children’s negative emotions. Furthermore, children’s negative emotions were significantly and negatively related to their tendency to play as usual (β = −0.377; *p* = 0.042; BootLLCI = −0.768; BootULCI = −0.024), indicating that high levels of children’s negative emotions were associated with a lower propensity to play independently. For Model 2, the total mediation effect of parental distress on children’s adaptive behavior mediated by children’s negative emotions was β = −0.638, *p* = 0.004 (BootLLCI = 1.068; BootULCI = −0.208). Finally, except for children’s sport practice during the lockdown (β = −0.251; *p <* 0.001; BootLLCI = −0.385; BootULCI = −0.291)—which was significantly and negatively related to the children’s negative emotions—all other covariates were not significantly associated with children’s negative emotions or adaptive behavior.

### 4.2. Comparison between before and during Lockdown in the ASD Sample

To explore children’s behavioral problems before and during the lockdown, paired sample *t*-tests were carried out. The results showed significant differences regarding parental perception of children’s stereotypes (*t*(52) = −3.476, *p* = 0.001) and repetitive behaviors (*t*(52) = −2.793, *p* = 0.007): children showed more behavioral problems during the lockdown (stereotypes: M = 4.30; sd = 1.99; repetitive behaviors: M = 2.64; sd = 2.26) than before it (stereotypes: M = 3.68; sd = 1.99; repetitive behaviors: M = 2.06; sd = 1.95). Regarding children’s aggressive behaviors (towards the self and others) and the transition from one activity to another, no significant differences were found. 

### 4.3. Comparison between Parental Distress and Children’s Outcomes in ASD and TD Children

Spearman’s correlations carried out on the TD sample showed similar results to those found in the ASD sample. Specifically, parental distress was significantly and negatively correlated with children’s positive emotions (*r* = −0.324; *p* = 0.008) and playing activities (*r* = −0.523; *p* < 0.001); this means that high distress levels indicate lower parental ability to perceive children’s positive emotions and their ability to play as usual during the lockdown. Parental distress was significantly and positively correlated with their children’s negative emotions (*r* = 0.500; *p <* 0.001); in other words, distressed parents perceived their children as being sad, angry, worried, and anxious during the lockdown. The expected correlations emerged between children’s positive (*r* = 0.345; *p* = 0.005) and negative (*r* = −0.331; *p* = 0.007) emotions, and their adaptive behavior, i.e., playing as usual.

Furthermore, independent *t*-tests showed significant differences between the two groups of children in the majority of the variables considered. Specifically, results (*t*(95) = −3.166; *p* = 0.002) showed that parents of children with ASD (M = 1.89; sd = 0.54) were more distressed than parents of TD children (M = 1.61; sd = 0.41). Furthermore, results indicated that TD children (M =3.62; sd = 0.89) were perceived as more independent during playing activities (*t*(117) = 4.041; *p* < 0.001) than ASD children (M = 2.96; sd = 0.88). Regarding children’s emotional responses, the analyses did not reveal any significant differences. Therefore, we investigated whether there were any differences between the two groups in each child’s positive and negative emotions as perceived by the parent(s). Specifically, findings showed that ASD children reached higher scores on happy (M = 3.45; sd = 0.91) and anxious (M = 2.68; sd = 0.94) emotions than TD children (happy: M = 2.97; sd = 0.84; anxious: M = 2.14; sd = 0.99) during the social isolation imposed by COVID-19. 

### 4.4. Mediation Models with Group (ASD vs. TD) as a Covariate 

**Figure 3:**
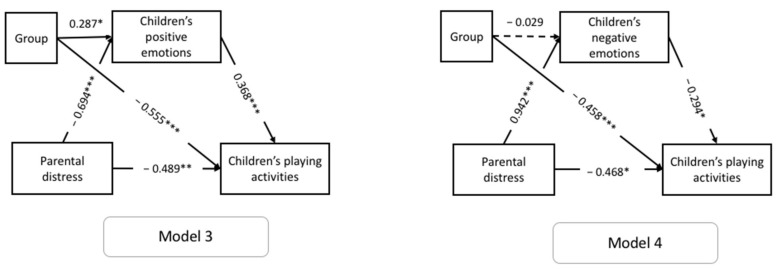
Model 3 and 4 testing the expected mediation relationships, with the group as a covariate. * *p* < 0.05; ** *p* < 0.01; *** *p* < 0.001.

To evaluate whether there were any differences between ASD and TD children in the hypothesized tested models, the two models were re-tested with the group as a covariate. Model 3 included children’s positive emotions as mediators, and Model 4 included children’s negative emotions. Figure 3 shows the models tested.

Regarding Model 3 (F(2, 116) = 12.582; *p* < 0.001; R = 0.51; R^2^ = 0.26), parental distress was significantly and negatively related to children’s tendency to play (β = −0.489; *p* = 0.005; BootLLCI = −0.831; BootULCI = −0.149) and to their positive emotions (β = −0.694; *p* < 0.001; BootLLCI = −0.983; BootULCI = −0.371), indicating that increased parental distress levels were associated with children’s lower ability to play as usual, and with their decreased positive emotions. Furthermore, children’s positive emotions were significantly and positively related to their adaptive behavior (β = 0.368; *p* < 0.001; BootLLCI = 0.169; BootULCI = 0.558), indicating that children’s increased positive emotions were associated with their greater independence to play. For Model 3, the total effect of parental distress on children’s adaptive behavior mediated by children’s negative emotions was β = −0.744, *p <* 0.001 (BootLLCI = 1.064; BootULCI = −0.425). Finally, results revealed a significant impact of the group (ASD vs. TD) on the mediator and the children’s outcomes: parents with ASD children perceived their positive emotions more (β = 0.287; *p* = 0.037; BootLLCI = 0.035; BootULCI =.534), and their ability to play as usual as lower (β = −0.555; *p <* 0.001; BootLLCI = −0.848; BootULCI = −0.253), than parents of typically developing children. 

Regarding Model 4 (F(2, 116) = 20.231; *p* < 0.001; R = 0.51; R^2^ = 0.26), parental distress was significantly and negatively related to the children’s tendency to play (β = −0.468; *p* = 0.016; BootLLCI = −0.842; BootULCI = −0.091), indicating that increased parental distress levels were associated with children’s lower ability to play. Distress was significantly and positively related to children’s negative emotions (β = 0.942; *p* < 0.001; BootLLCI = 0.631; BootULCI = 1.217), indicating that increased parental distress levels were associated with children’s greater negative emotions. Furthermore, children’s negative emotions were significantly and negatively related to their adaptive behavior (β = −0.294; *p* = 0.013; BootLLCI = −0.499; BootULCI = −0.090), indicating that children’s increased negative emotions were associated with children’s lower independence to play. For Model 4, the total effect of parental distress on children’s adaptive behavior mediated by children’s negative emotions was β = −0.744, *p* < 0.001 (BootLLCI = 1.064; BootULCI = −0.425). Finally, results revealed a significant impact of the group (ASD vs. TD) on the children’s outcomes: parents with ASD children perceived their children’s ability to play as lower (β = −0.458; *p* = 0.004; BootLLCI = −0.762; BootULCI = −0.154) than parents of typically developing children.

## 5. Discussion and Future Directions

The COVID-19 outbreak led researchers to devote their attention to its impact on the caregiving system. Specifically, a huge body of literature examined the impact of public health measures (e.g., lockdown, quarantine, social isolation, etc.) on families with typically developing children [34], vulnerable children [40,41], or autistic children [46,47,49]. Our study aimed at contributing to this field of research, focusing on families with children with ASD. It was already well known that the behaviors of children with autism are negatively affected by disruption to their daily life routines [37,55]. Therefore, it was worth examining the impact on these families of the lockdown, of social isolation, and of the lack of school- and therapeutic-related activities because of the COVID-19 pandemic.

The purpose of the present study was to investigate the interplay between parental distress, children’s emotional responses, and their adaptive behavior (i.e., playing activities as usual) experienced during the lockdown. Specifically, we explored this interplay in a sample of families with ASD children aged 5–10 years, hypothesizing (HP1) that parental distress may affect children’s adaptive behavior through the mediation role of children’s positive (Model 1) and negative (Model 2) emotional responses, as perceived by parents. We included children’s ability to play as usual and independently as an example of adaptive behavior, because it is a global index of the children’s wellbeing and adjustment [56]. Furthermore, we investigated in these families the possible impact of the lockdown on children’s behavioral problems, comparing them before and during the lockdown (RQ1). To the best of our knowledge, there were no studies comparing families with a ASD and TD children in terms of these psychological outcomes. Therefore, we firstly compared these two groups in terms of parental distress levels, children’s emotional responses, and children’s adaptive behavior (RQ2); secondly, we tested the hypothesized mediation models (Model 1 and Model 2) on these two groups (Model 3 and Model 4) (RQ3). 

### 5.1. The Interplay between Parental Distress and Children’s Adaptive Behavior

The findings of the tested models corroborated our HP1 and confirmed parental distress as a risk factor that could influence parents’ perception of children’s emotion regulation and adaptive behaviors. In both models (Model 1 and 2), we found that parental distress influenced the children’s tendency to play independently through the mediation of their emotions. Higher distress led to lower positive and higher negative emotions. Positive and negative emotions, in turn, influenced playing activities positively and negatively, respectively. According to Morris et al.’s [50] tripartite model, our results supported the idea that parental distress negatively impacts the caregiver-child relationship [57,58]. High levels of parental distress negatively influence the family dynamics and the emotional environment [59], and this, in turn, negatively affects children’s emotional regulation [60,61] and their adaptive behaviors [62]. Furthermore, when parents show high distress levels, their ability to recognize and correctly interpret the children’s emotional responses could be impaired [61], and they may perceive the children’s behavior as more difficult to manage [63]. These negative relationships may be amplified in cases of atypical development conditions (e.g., children with an ASD), because parents are already in a stressful condition. 

We also explored the role of several other factors (e.g., children’s gender, age, and functioning; the number of professionals supporting the child; frequency of sport practice) on children’s emotional responses and on their ability to play as usual. Specifically, for Model 1, we found that children’s functioning and their sport training significantly impacted on children’s positive emotions. In other words, parents of low-functioning children perceived fewer positive emotions in their children compared to parents with children with high-functioning autism. This result has to be considered in the light of the fact that children with low-functioning autism experience difficulty in their ability to express emotions [64,65,66]. Regarding sport practice during the lockdown, the results highlighted that the more sport activities were conducted during the lockdown, the higher were the positive emotions (Model 1), and the lower the negative ones (Model 2). These results are consistent with other recent evidence [67] on the positive effects of sport training during the lockdown, suggesting the potential protective role of physical activity and sport during a stressful situation, such as the COVID-19 lockdown. Participation in physical activities contributes to the reduction of children’s stress levels [68], and this may be linked to the expression of more positive emotions and less negative ones, as found in our study.

### 5.2. Stability of Behavioral Problems before and during the Lockdown

The disruption of the daily life routine, especially for children with ASD, negatively impacted on their behavioral problems [37,44]. Therefore, we explored this topic in our study. Our results showed an increase in the stereotyped and repetitive behaviors during the lockdown compared to the period before the pandemic. These findings are consistent with other studies [48,69], which found that children’s behavioral problems increased, were more intense, and were more frequent during the lockdown.

### 5.3. Comparison between ASD and TD Children

The present research also tested whether there were any differences between families with children with ASD and families with children under typical developmental conditions in terms of parental distress, children’s emotional responses, and children’s adaptive behavior. We firstly compared the two groups of children (RQ2), and then we investigated whether there were any differences in the tested models, considering the group as a covariate (autism vs. typical development) (RQ3). The comparisons showed that parents of children with ASD were more distressed than those with typically developing children. The disruption to school- and therapeutic-related activities because of the lockdown forced parents to take care of their children alone, holding different roles at the same time (teacher or therapist), for which they were not coached, as well as simultaneously having to handle familial and work requirements. Moreover, parents of children with ASD perceived them as less independent in play compared to parents of TD children, as found in the absence of stressful conditions. Because results showed no significant differences between ASD and TD children in terms of their positive and negative emotional responses as aggregate variables, we compared the two groups based on each emotion evaluated by parents. The findings showed that parents of children with an ASD perceived them as happier and more anxious than parents of TD children. 

Finally, we also explored whether there were any differences in the hypothesized models according to the children’s group (RQ3). In both Models 3 and 4, we found direct effects of parental distress on children’s adaptive behavior (playing activities), and mediated effects of the children’s emotional responses on the previous relationship. These results confirmed what emerged from Models 1 and 2: the effect of the group, as a covariate, corroborated the findings of the comparisons between children with an ASD and typically developing children. Parents in the ASD group had children expressing more positive emotions, but less independent to play compared to parents of TD children.

The present study has several limitations. Our study was cross-sectional; therefore, no causal considerations can be drawn. To understand and delineate the impact that the COVID-19 outbreak may have had on the behavioral and emotional functioning of individuals with ASD and their families, follow-up studies with a longitudinal component are needed. We also hypothesized that parental distress would be related to children’s adaptive behaviors via the mediation of children’s emotional responses. One may argue that, in clinical practice, parenting distress often results from their children’s behavioral and emotional problems. Considering such clinical evidence, further studies should consider these kinds of reverse relationships, measuring both behavioral and emotional problems that were not considered in the present paper. Moreover, we considered children from 5 to 10 years—further studies on different age groups (e.g., adolescents or young adults) are especially necessary. Further studies should also try to match TD children and ASD children, at least in terms of their chronological age. Third, the measures applied are not fully validated, although they have been applied elsewhere with comparable results [34,53]. Fourth, the online survey collected data mainly regarding male children with ASD; thus, we had less information regarding the impact of the COVID-19 outbreak on female children with ASD. This topic on the relationship between the COVID-19 outbreak and female individuals with ASD was poorly investigated in the current literature, probably because of the gender ratio of autism. Further studies are needed in this vein, because only one study [70] investigating this relationship considered both female and male adult individuals, and reported poorer coping strategies and a greater overall negative impact in adult females compared to peer males. Therefore, future studies could investigate this relationship by comparing both genders.

Fifth, the inclusion of control groups with other clinical conditions (i.e., intellectual disabilities without ASD, epilepsy, diabetes, etc.) may further support the specificity of our findings. Finally, all the measures are parent-reported. Although this represents a limitation, due to the desirability bias, involving children with ASD during a lockdown was not possible.

## 6. Conclusions and Future Directions

The present paper devotes special attention to ASD, which has been a deeply studied psychiatric condition in the past few years, in terms of early screening [71,72,73], diagnosis [74,75] and intervention [76]. Nevertheless, the COVID-19 pandemic emergency has forced researchers to refocus their efforts on unique relevant issues concerning the psychological support of children with ASD and their families during this stressful time, in order to help them in managing their distress and their disrupted daily life routines. During the COVID-19 pandemic, several studies have been published with the aim of supporting both professionals [77] and parents [37,47] in managing children’s behaviors and routines. The outbreak exacerbated the distress of families of children with ASD and increased the children’s stereotyped and repetitive behaviors compared to the period before. Indeed, our findings highlighted that, during the lockdown, positive and negative emotions—as reported by parents—mediated the relationship between parental distress and children’s adaptive behavior. In this line, our results may provide further knowledge to inform online-based parent-coaching interventions. The intervention programs could include suggestions for parents regarding how to better interact and play with their children, and how to better communicate with them [37]. These kinds of interventions may reduce distress and help in developing adequate coping strategies [78], and at the same time, they may have an indirect effect on children’s adaptive behavior via their emotional regulation. 

Finally, in order to broaden one of the topics investigated in the present paper—specifically, the impact of a new event (i.e., the COVID-19 pandemic) on the routines of children with autism aged 5–10 years—it could be interesting to elaborate future studies investigating the impact of the commencement of school activities—which represents a remarkable event in childhood—during the COVID-19 outbreak on the children’s routines and familial management.

## Figures and Tables

**Table 1:** Description of the socio-demographic variables of the ASD and TD samples, and results of the comparisons.

	ASD (*n* = 53)	TD (*n* = 67)	Comparisons
Children’s gender—*n*	43 males	55 males	χ^2^ (1) = 018; *p* = 0.893
Children’s age—M (sd)	6.94 (1.6)	8.47 (1.04)	*t*(83) = 5.843; *p* <0 001
Children’s functioning—*n*			
High-functioning	26	-	-
Low-functioning	27		
Parental exposure to COVID—M (sd)	0.45 (0.66)	0.45 (0.82)	*t*(98) = −1.401; *p* = 0.164
Parents’ age—M (sd)	41.8 (5.4)	40.2 (6.5)	*t*(117) = −1.373; *p* = 0.172
Parents’ status—%			
Married/with partner	88.7%	88.1%	χ^2^ (2) = 1.170; *p* = 0.552
Divorced/separated	7.5%	4.5%	
Single	3.8%	7.5%	
Parents’ educational level ^1^—%			
Low	7.5%	13.4%	χ^2^(2) = 2.460; *p* = 0.292
Intermediate	41.5%	34.3%	
High	50.9%	52.2%	
Parents’ occupational status—%			
Employed	73.6%	68.7%	χ^2^ (2) = 6.086; *p* = 0.298
Not employed	3.8%	3.8%	
Housekeeper	22.6%	22.6%	
Online learning condition—%			
Class lessons	30.2%	-	-
Individual lessons	22.6%		
Mixed condition (class and individual lessons)	17%		
No lessons ^2^	30.2%		
Level of children’s difficulty in doing homework—parent-reported—%			
No difficulty at all	7.5%	-	-
Low difficulty	18.9%		
Intermediate difficulty	24.5%		
High difficulty	18.9%		
Number of professionals who supported the families—%			
None	26.4%	-	-
One	54.7%		
Two	18.9%		
Home balcony—%			
Yes	83%	-	-
No	17%		
Special certification from children’s neuropsychiatrist ^3^—%			
Yes	24.5%	-	-
No	75.5%		
Frequency of sport practice			
No practise at all	30.2%	-	-
Less than usual	39.6%		
As usual	7.5%		
More than usual	7.5%		

Note: ^1^ low = up to 13 years, corresponding to junior high school; intermediate = up to 18 years, corresponding to high school; high = more than 15 years, corresponding to university degree or beyond. ^2^ 30.2% corresponds to 16 children; 6 of them were 5 years old, the others were 6 or older. ^3^ The certification from the children’s neuropsychiatrist was a special permission, with particular attention to families with ASD children, to go out for a walk despite the lockdown.

## Data Availability

Data are available from the first author upon reasonable request.
